# Hyperglycemia and Correlated High Levels of Inflammation Have a Positive Relationship with the Severity of Coronavirus Disease 2019

**DOI:** 10.1155/2021/8812304

**Published:** 2021-03-18

**Authors:** Wen Zhang, Chuanwei Li, Yu Xu, Binfeng He, Mingdong Hu, Guoqiang Cao, Li Li, Shuang Wu, Xia Wang, Chun Zhang, Jianping Zhao, Jungang Xie, Zihui Xu, Qi Li, Guansong Wang

**Affiliations:** ^1^Institute of Respiratory Diseases, Department of Pulmonary and Critical Care Medicine, Xinqiao Hospital, Third Military Medical University, Chongqing 400037, China; ^2^Infection Division, Wuhan Huoshenshan Hospital, Wuhan 430030, China; ^3^Infection Division, Wuhan Jinyintan Hospital, Wuhan 430030, China; ^4^Department of Cardiology, Daping Hospital, Third Military Medical University, Chongqing 400037, China; ^5^Department of Critical Care Medicine, Wuhan Huoshenshan Hospital, Wuhan 430030, China; ^6^Department of Pulmonary and Critical Care Medicine, Daping Hospital, Third Military Medical University, Chongqing 400037, China; ^7^Department of Pulmonary and Critical Care Medicine, Tongji Hospital, Tongji Medical College, Huazhong University of Science and Technology, Wuhan 430030, China; ^8^Department of Traditional Chinese Medicine, Xinqiao Hospital, Third Military Medical University, Chongqing 400037, China

## Abstract

**Objective:**

Coronavirus disease 2019 (COVID-19) is a considerable global public health threat. This study sought to investigate whether blood glucose (BG) levels or comorbid diabetes are associated with inflammatory status and disease severity in patients with COVID-19.

**Methods:**

In this retrospective cohort study, the clinical and biochemical characteristics of COVID-19 patients with or without diabetes were compared. The relationship among severity of COVID-19, inflammatory status, and diabetes or hyperglycemia was analyzed. The severity of COVID-19 in all patients was determined according to the diagnostic and treatment guidelines issued by the Chinese National Health Committee (7th edition).

**Results:**

Four hundred and sixty-one patients were enrolled in our study, and 71.58% of patients with diabetes and 13.03% of patients without diabetes had hyperglycemia. Compared with patients without diabetes (*n* = 366), patients with diabetes (*n* = 95) had a higher leucocyte count, neutrophil count, neutrophil to lymphocyte ratio (NLR), and erythrocyte sedimentation rate (ESR). There was no association between severity of COVID-19 and known diabetes adjusted for age, sex, body mass index (BMI), known hypertension, and coronary heart disease. The leucocyte count, NLR, and C-reactive protein (CRP) level increased with increasing BG level. Hyperglycemia was an independent predictor of critical (OR 4.00, 95% CI 1.72-9.30) or severe (OR 3.55, 95% CI 1.47-8.58) COVID-19, and of increased inflammatory levels (high leucocyte count (OR 4.26, 95% CI 1.65-10.97), NLR (OR 2.76, 95% CI 1.24-6.10), and CRP level (OR 2.49, 95% CI 1.19-5.23)), after adjustment for age, sex, BMI, severity of illness, and known diabetes.

**Conclusion:**

Hyperglycemia was positively correlated with higher inflammation levels and more severe illness, and it is a risk factor for the increased severity of COVID-19. The initial measurement of plasma glucose levels after hospitalization may help identify a subset of patients who are predisposed to a worse clinical course.

## 1. Introduction

The outbreak of coronavirus disease 2019 (COVID-19) was first identified in Wuhan, Hubei Province, China, at the end of 2019 [1, 2]. By early May 2020, more than 84,000 cases had been confirmed, almost 4,600 people had died, and 79,194 cases had been cured in China. Due to the substantial efforts across the country, the number of reported cases of COVID-19 has fallen dramatically to only a few sporadic cases. However, 11,500,302 confirmed cases have been reported globally, with more than 535,000 deaths to date [3]. COVID-19 is a serious global public health threat.

Diabetes and hyperglycemia are of interest because they are associated with poor outcomes of acute medical conditions among hospitalized patients, including COVID-19. For 233 patients with pneumococcal pneumonia, 30-day mortality among those with admitting blood glucose (BG) ≥ 10 mmol/L was 3.4 times (95% confidence interval (CI) 0.85-13.40) greater than among those with admitting BG < 7 mmol/L. There was also a strong association between the disease severity and admitting BG among patients without diabetes [4]. Of the 2,471 patients with community-acquired pneumonia, 279 (11%) patients had BG > 11 mmol/L on admission. Patients with hyperglycemia (BG > 11 mmol/L) had an increased risk of death (13 vs. 9%) and in-hospital complications (29 vs. 22%) compared with those with BG ≤ 11 mmol/L [5]. Furthermore, hyperglycemia (BG ≥ 7.0 mmol/L) (odds ratio (OR) 3.3, 95% CI 1.4-7.7) and comorbid diabetes (OR 3.0, 95% CI 1.4-6.3) were independent predictors of mortality in patients with severe acute respiratory syndrome (SARS) [6]. Diabetes (OR 2.6, 95% CI 1.4-4.9) was the primary comorbidity associated with mortality of MERS-CoV infections [7].

Recent studies reported that COVID-19 patients with comorbidities were at increased risk of mortality [8, 9], and diabetes is one of the most common comorbidities in COVID-19 patients [10, 11]. Early studies showed that 7.4-19% of patients with COVID-19 had comorbid diabetes, and the proportion of patients with diabetes was higher (16.2-26.9%) among patients with severe COVID-19 [8, 12, 13]. These COVID-19 patients (*n* = 451) with diabetes and/or uncontrolled hyperglycemia (BG > 10 mmol/L) had a longer length of stay (5.7 vs. 4.3 days) and markedly higher mortality (28.8 vs. 6.2%) than patients without diabetes or uncontrolled hyperglycemia [14]. Nevertheless, previous published reports found no association between adverse outcome and glucose control (BG 4.4 to 6.1 mmol/L) at the time of admission for critically ill patients [15]. Therefore, whether the degree to which hyperglycemia before the administration of steroids is associated with adverse outcomes of COVID-19 is still under debate.

In this study, we investigated diabetic and nondiabetic patients with laboratory-confirmed COVID-19 who were admitted to Wuhan Jinyintan Hospital, Huoshenshan Hospital and Tongji Hospital. Determining the baseline severity of COVID-19, inflammatory status and levels of average BG before the administration of steroids in patients with and without diabetes will facilitate a better understanding of hyperglycemia and diabetes in patients with COVID-19.

## 2. Methods

### 2.1. Study Design and Participants

This multicenter, retrospective, observational study was performed at Wuhan Jinyintan Hospital, Huoshenshan Hospital, and Tongji Hospital (Wuhan, China), which are hospitals designated for the treatment of patients with COVID-19. We retrospectively analyzed patients from January 28, 2020, to March 2, 2020, who had been diagnosed with COVID-19 according to the World Health Organization (WHO) interim guidance [16]. Laboratory confirmation of severe acute respiratory syndrome coronavirus 2 (SARS-CoV-2) infection was performed by the local health authority as previously described [1, 2]. The presence of SARS-CoV-2 in respiratory specimens was detected by next-generation sequencing or real-time RT-PCR methods. Patients lacking or with negative SARS-CoV-2 test results were excluded from this study. All patients involved in this study were living in Wuhan during the outbreak of COVID-19. The Ethics Commission of Huoshenshan Hospital approved this study. The need for written informed consent was waived due to the rapid emergence of this infectious disease.

### 2.2. Data Collection

Demographic information, clinical characteristics (including symptoms, medical history, and comorbidities), chest computed tomography (CT) scans, and laboratory findings for each patient were obtained from the electronic medical record system of Wuhan Jinyintan Hospital, Huoshenshan Hospital, and Tongji Hospital and analyzed by three independent researchers. Patients with diabetes and other coexisting medical conditions (including cerebrovascular disease, endocrine system disease, malignant tumor, respiratory system disease, and nervous system disease) were identified. The dates of disease onset and hospital admission and the severity of COVID-19 were also recorded.

The severity of COVID-19 and treatment for all patients were defined according to the diagnostic and treatment guidelines for COVID-19 issued by the Chinese National Health Committee (7th edition) [17].

Severe COVID-19 was diagnosed when the patients met one of the following criteria: (a) respiratory distress with respiratory frequency ≥ 30 breaths/min; (b) pulse oximeter oxygen saturation level ≤ 93% at rest; or (c) oxygenation index (arterial partial pressure of oxygen/inspired oxygen fraction, PaO2/FiO2) ≤ 300 mmHg.

Critical COVID-19 was diagnosed when the patients met one of the following criteria: (a) respiratory failure requiring mechanical ventilation; (b) septic shock; or (c) other organ failure necessitating intensive care unit monitoring and treatment.

Hyperglycemia was defined as any glucose value greater than 7.8 mmol/L (140 mg/dL) on 2 or more determinations prior to initial corticosteroid use during hospitalization [18]. Fasting blood glucose (FBG) test was conducted by a laboratory plasma measurement. Random BG test of critically ill patients was conducted by a bedside capillary measurement, and increased random BG during intravenous dextrose infusion was excluded according to nursing records. The frequency of fasting and random BG varied between individuals, depending on the severity of hyperglycemia. FBG was tested every 5-7 days for patients without diabetes. The BG level of patients with diabetes and hyperglycemia in intensive care unit was tested every 4-6 hours. The mean BG is calculated as the sum of all BG divided by test number for each patient.

### 2.3. Statistical Analysis

In the previous studies about COVID-19 [13, 19], the probability of being severe and critical illness for nondiabetics was 0.178, the probability of being severe and critical illness for diabetics was 0.427, and the percentage of patients with known diabetes in all COVID-19 patients was 7.92%. We calculated that we would need to enroll 440 patients at least to have a power of 90% to detect the relationship between diabetes and severity of illness using the PASS version 11, at an alpha level of 0.05 (two-tailed).

The measurement data are expressed as the mean ± SD or median (interquartile range, IQR), as appropriate. The enumeration data are reported as percentages or *n* (%). Mean values and percentages were compared among different groups by ANOVA and *χ*^2^ tests or Fisher's exact test, as appropriate. Nonnormally distributed values were compared between two groups using the Mann–Whitney *U* test and among more than two groups using the Kruskal-Wallis test. Correlations between FPG levels and the severity of COVID-19, hospitalization duration, and levels of inflammatory markers were assessed with Spearman's correlation. The association of the severity of COVID-19 with known diabetes and plasma glucose levels was assessed with multivariable logistic regression analysis. All data were analyzed using IBM SPSS version 19.0 (SPSS, Chicago, IL, USA). *P* < 0.05 (two-tailed) was considered statistically significant.

## 3. Results

### 3.1. Patient Characteristics and Laboratory Parameters

Four hundred and sixty-one patients with COVID-19 were included in this study. Most patients were middle- and old-aged (63.0 [IQR 54.0-69.0]), with roughly equal proportions of men and women (52.1% vs. 47.9%). Known diabetes was one of the top three comorbidities, and critical patients were more likely than patients with less severe COVID-19 to have comorbid diabetes (32.0% vs. 26.3% and 17.0% in the critical, severe, and general group, respectively, *P* = 0.008). On admission, fever (374 [81.1%]) and cough (359 [77.9%]) were the most common symptoms. More than half of the patients (265 [57.5%]) developed dyspnea, and the proportions were higher in the severe and critical groups (45.6% vs. 82.5% and 90.7% in the general, severe, and critical groups, respectively, *P* < 0.001). The mean hospitalization duration was almost two weeks, but it was as long as three weeks in the critical group.

The leucocyte and neutrophil counts were the highest, while the lymphocyte counts were the lowest in the critical group (median leucocytes 9.20 × 10^9^/L [IQR 6.59-14.10]; median neutrophils 8.18 × 10^9^/L [IQR 5.27-12.86]; and median lymphocytes 0.57 × 10^9^/L [IQR 0.36-0.85]) compared with the general and severe groups (Table 1). Thus, the neutrophil to lymphocyte ratio (NLR) and platelet to lymphocyte ratio (PLR) (median NLR 15.2 [IQR 8.08-28.57]; median PLR 293.81 [IQR 155.21-430.03]) were significantly higher in critical patients. Consistent with this finding, the procalcitonin (PCT) level, C-reactive protein (CRP) level, and erythrocyte sedimentation rate (ESR) were markedly higher in the critical group (median PCT 0.24 ng/mL [IQR 0.11-0.60], median CRP 93.40 mg/L [IQR 39.42-159.10], and median ESR 44.0 mm/h [IQR 39.0-92.0], respectively), indicating that critical patients suffered from the most severe inflammatory response.

The mean BG level before the administration of steroids increased with the increasing severity of COVID-19, with values of 5.70 mmol/L (IQR 4.90-6.95) in the general group, 6.63 mmol/L (IQR 5.04-8.68) in the severe group, and 8.10 mmol/L (IQR 6.25-10.60) in the critical group. In total, 114/448 (25.4%) patients had hyperglycemia (BG > 7.8 mmol/L), and the prevalence of hyperglycemia was the highest in the critical group (52.0% vs. 17.4%, 35.7% in the critical, general, and severe groups, respectively, *P* < 0.001). Glycated hemoglobin (HbA1c) (*n* = 88) ranged from 5.5 to 12.2% with a median of 6.0%. Ninety-five of 461 patients had a known history of diabetes (20.61%), with 56 (58.95%) patients on only insulin therapy, 25 (26.32%) on oral antidiabetic agents (including metformin, sulfonylureas and dipeptidyl peptidase 4-inhibitors), and 11 (11.58%) on a combination of insulin and orals. Five and zero patients had level 1 hypoglycemia (3.0-3.9 mmol/L) on admission and during hospitalization, respectively. No patient had acute hyperglycemic crises (such as diabetic ketoacidosis or hyperosmolar hyperglycemic state) on admission or during hospitalization. Patients were prescribed subcutaneous fast acting and/or long acting insulin as per hospital protocol.

### 3.2. COVID-19 Patients with Known Diabetes

We found that the prevalence of known diabetes in the critical patients was much higher than the prevalences in the general and severe patients. To explore the clinical characteristics of COVID-19 patients with known diabetes, we analyzed the data grouped by the diagnosis of diabetes and stratified by hyperglycemia or normoglycemia (Table 2). The mean BG levels before the administration of steroids were markedly higher in the diabetic group than that in the nondiabetic group (9.91 mmol/L [IQR 7.69-12.30] vs. 5.54 mmol/L [IQR 4.90-6.65]; *P* < 0.001). Sixty-eight (71.58%) patients with diabetes suffered from hyperglycemia, while 46 (13.03%) patients without diabetes suffered from hyperglycemia.

There was a higher proportion of critical patients in the diabetic group than that in the nondiabetic group (25.3% vs. 13.9%; *P* = 0.008). The duration of hospitalization in the diabetic group was much longer than that in the nondiabetic group (16.0 [IQR 14.0-18.0] days vs. 12.0 [IQR 10.0-14.0] days; *P* < 0.001). Compared with the nondiabetic group, the diabetic group had a higher leucocyte count (6.59 × 10^9^/L [IQR 4.80-9.00] vs. 5.58 × 10^9^/L [IQR 4.39-7.50]; *P* = 0.004), neutrophil count (4.78 × 10^9^/L [IQR 3.21-6.95] vs. 3.85 × 10^9^/L [IQR 2.78-5.51]; *P* = 0.003), NLR (4.39 [IQR 2.47-10.01] vs. 3.22 [IQR 2.00-5.90]; *P* = 0.022), and ESR (48.3 mm/h [IQR 38.0-64.1] vs. 37.5 mm/h [IQR 19.0-61.5]; *P* = 0.038). After the stratified analysis by hyperglycemia or normoglycemia, we found that patients with hyperglycemia, whether they were in the diabetic or nondiabetic group, had more severe COVID-19, a greater inflammatory response, and a longer hospital stay than those with normoglycemia.

Furthermore, we conducted multiple ordinal logistic regression analysis to assess the relationship between known diabetes and the severity of COVID-19. There was no association between severity of COVID-19 and known diabetes after adjustment for age, sex, BMI, known hypertension, and coronary heart disease (OR 1.39, 95% CI 0.64-3.03; *P* = 0.406 for critical illness. OR 1.54, 95% CI 0.66-3.59; *P* = 0.316 for severe illness). However, known diabetes was an independent predictor of a high leucocyte count (>10 × 10^9^/L) (OR 2.05, 95% CI 1.04-4.05; *P* = 0.039) and high ESR (>30 mm/h) (OR 5.9, 95% CI 1.2-28.9; *P* = 0.030) in COVID-19 patients after adjustment for age, sex, and severity of illness. In addition, a known history of diabetes was an independent predictor of a longer hospitalization duration (OR 7.7, 95% CI 3.1-18.9; *P* < 0.001) after adjustment for age, sex, and severity of illness.

### 3.3. Severity, Inflammation Level, and BG

Before the initiation of steroid treatment, mean BG levels on admission were significantly higher in the critical group than in the general and severe groups in Table 1. Thus, we conducted multivariable ordinal logistic regression analysis to discover whether there was a relationship between BG levels and the severity of COVID-19. Hyperglycemia was an independent predictor of having critical (OR 4.00, 95% CI 1.72-9.30; *P* = 0.001) and severe (OR 3.55, 95% CI 1.47-8.58; *P* = 0.005) COVID-19 after adjustment for age, sex, BMI, a known history of diabetes, hypertension, and coronary heart disease. For each 1 mmol/L increase in the blood glucose level, the absolute risk of having critical COVID-19 increased by 25.7% (95% CI 10.7-42.8; *P* < 0.001).

**Figure 1:**
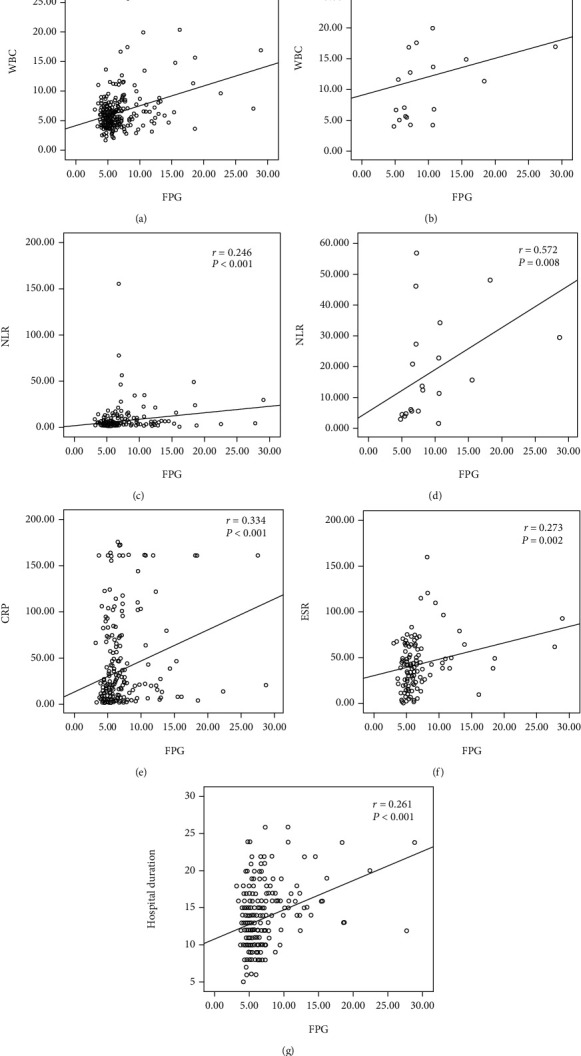
Correlations between the levels of inflammatory markers, hospitalization duration, and FPG levels from COVID-19 patients. (a, c, e, f, g) For all patients. (b, d) For the critical group. Spearman's test was used to evaluate the correlation. WBC: white blood cell count; NLR: neutrophil to lymphocyte ratio; CRP: C-reactive protein; ESR: erythrocyte sedimentation rate; FPG: fasting plasma glucose; COVID-19: coronavirus disease 2019.

Similar correlations were also found between FBG levels and inflammatory markers, including the NLR (*r* = 0.246, *P* < 0.001), CRP level (*r* = 0.334, *P* < 0.001), and ESR (*r* = 0.273, *P* = 0.002) (Figure 1). In the subgroup analysis stratified by severity of illness, more obvious positive correlations were found between FBG levels and the leucocyte count (*r* = 0.454, *P* = 0.044) and between FBG levels and the NLR (*r* = 0.572, *P* = 0.008) in the critical group than that in the other groups. After the multivariable logistic regression analysis was conducted, we discovered a relationship between BG levels and the level of inflammation. Hyperglycemia was an independent predictor of a high leucocyte count (>10 × 10^9^/L) (OR 4.26, 95% CI 1.65-10.97; *P* = 0.003), NLR (>5) (OR 2.76, 95% CI 1.24-6.10; *P* = 0.013), and CRP level (>15 mg/L) (OR 2.49, 95% CI 1.19-5.23; *P* = 0.016) in patients with COVID-19 after adjustment for age, sex, BMI, severity of illness, and a known history of diabetes. For each 1 mmol/L increase in the blood glucose level, the absolute risk of a high WBC count increased by 32.2% (95% CI 14.6-52.5; *P* < 0.001), the absolute risk of a high NLR increased by 18.3% (95% CI 5.6-32.7; *P* = 0.004), and the absolute risk of a high CRP level increased by 15.2% (95% CI 2.5-29.5; *P* = 0.018).

## 4. Discussion

Here, we report a multicenter, retrospective, observational study of 461 patients with laboratory-confirmed COVID-19. Hyperglycemia was an independent predictor of the level of inflammation and the severity of COVID-19. Patients with hyperglycemia, irrespectively of the presence of overt diabetes, were at significantly higher risk of suffering from severe COVID-19 and of having a great inflammatory response than those with normoglycemia.

In our study, 95 (20.61%) patients had a known history of diabetes, and the proportion of patients with diabetes was as high as 32.00% in the critical group. This rate was similar in other studies focusing on COVID-19 patients with known diabetes [12, 13, 20]. Moreover, 68/95 (71.58%) patients with diabetes and 46/353 (13.03%) patients without diabetes had hyperglycemia while the levels of HbA1c on admission were nearly normal in both groups. This result indicated that patients with diabetes have a predisposition to BG fluctuation or poorly controlled BG when exposed to a virus. Moreover, 24.73% of patients had hyperglycemia in total, and 52.00% of critical patients had hyperglycemia in our study. Previous studies reported that 42.4-51% patients with COVID-19 had hyperglycemia (admission BG > 7.77 mmol/L) [15, 21], which was much higher than that in our study. This result may be due to the different proportions of disease severity in different studies, with the data of some studies obtained from moderate or severe patients [13, 22]. However, consistent with those studies, our study found almost half of critical patients had hyperglycemia, which should not be ignored when managing COVID-19 patients.

We found that plasma glucose levels, other than diabetes, was positively correlated with the severity of COVID-19. There was no association between severity of COVID-19 and a known history of diabetes after adjustment for age, sex, BMI, known hypertension, and coronary heart disease, which was different with some studies about COVID-19 with diabetes. Those studies, which stated diabetes in COVID-19 patients is associated with a two-fold increase in severity of COVID-19 [23], did not take some confounding factors into consideration. As each of these comorbidities (such as elderly age, hypertension, cardiovascular disease, and obesity) have been shown to be associated with severe COVID-19 [24, 25], the confounding factors would confuse the relationship of COVID-19 and diabetes. Thus, the different results need further research to confirm.

Hyperglycemia was an independent predictor of having critical (OR 4.00, 95% CI 1.72-9.30) and severe (OR 3.55, 95% CI 1.47-8.58) COVID-19 after adjustment for age, sex, BMI, a known history of diabetes, hypertension, and coronary heart disease. More patients with hyperglycemia belonged to the severe and critical groups than those with normoglycemia, regardless of their past history of diabetes. Similar results regarding the association of hyperglycemia and radiographic imaging of COVID-19 were also found [20], and well-controlled BG (3.9-10.0 mmol/L) was associated with markedly lower mortality compared to individuals with poorly controlled BG (>10.0 mmol/L) (adjusted HR, 0.14) during hospitalization [21]. Thus, hyperglycemia was considered a cause of poor outcome. However, no association was found between adverse outcome and strict glucose control (BG 4.4 to 6.1 mmol/L) for critically ill patients due to the increased hypoglycemia [15]. Therefore, hyperglycemia should be taken into consideration when judging the severity or prognosis of COVID-19 patients, but BG control in COVID-19 with hyperglycemia could be flexible.

Hyperglycemia was also an independent predictor of a high leucocyte count (OR 4.14, 95% CI 1.61-10.64), NLR (OR 3.23, 95% CI 1.49-7.04), and CRP level (OR 2.76, 95% CI 1.33-5.72) in patients with COVID-19. There were moderate positive correlations between FBG levels and leucocyte count (*r* = 0.454) and between FBG levels and NLR (*r* = 0.572) in the critical group. Thus, hyperglycemia is correlated with high levels of inflammation, which is consistent with other studies [26]. The massive release of cytokines and glucocorticosteroids during an overwhelming viral infection [11] induced the stimulation of gluconeogenesis and increased insulin resistance, as well as the possibility of acute pancreatic *β*-cell damage via angiotensin-converting enzyme 2 [6, 27], which might contribute to this elevation in blood glucose levels. Based on those results, it was suggested hyperglycemia would be a symptom of severe illness.

Our sample was large enough, and the power of the study was more than 80%, indicating our results are valid. However, the results may not be stable due to many related factors, and the reliability needs more studies to confirm. Although mortality information was not available in our study due to the rapid emergence of this infectious disease, BG level before the administration of steroids, the severity of COVID-19, and hospitalization duration were valuable metrics to determine the relationship of BG and severity of COVID-19. In addition, because of the increased neutrophil count and decreased lymphocyte count in patients with COVID-19, the NLR may be a good marker of infection.

## 5. Conclusions

In conclusion, our results showed that 24.73% of COVID-19 patients had hyperglycemia. More patients in the diabetic group (71.58 vs. 13.03%) had hyperglycemia during the course of COVID-19. Hyperglycemia was an independent predictor of high inflammation levels and severe COVID-19. The initial measurement of plasma glucose levels after hospitalization may help identify a subset of patients who are predisposed to a worse clinical course.

## Figures and Tables

**Table 1:** Demographics and laboratory findings of COVID-19 patients on admission to hospital.

	Normal range	All patients (*n* = 461)	General group (*n*=329)	Severe group (*n* = 57)	Critical group (*n* = 75)
BMI		23.40 (22.03-25.20)	23.38 (22.45-25.20)	22.48 (21.50-25.25)	23.42 (20.69-25.13)
WBC, ×10^9^/L	3.5-9.5	5.81 (4.50-7.80)	5.48 (4.20-6.96)	5.70 (4.61-8.85)∗	9.20 (6.59-14.10)∗∗††
<4	··	76 (16.5%)	64 (19.5%)	8 (14.0%)	4 (5.3%)
4–10	··	328 (71.1%)	253 (76.9%)	39 (68.4%)	36 (48.0%)
>10	··	57 (12.4%)	12 (3.6%)	10 (17.5%)	35 (46.7%)
NEU, ×10^9^/L	1.8-6.3	3.98 (2.82-5.82)	3.64 (2.61-4.87)	4.11 (3.11-6.15)∗	8.18 (5.27-12.86)∗∗††
LYM, ×10^9^/L	1.1-3.2	1.11 (0.75-1.50)	1.21 (0.90-1.66)	1.05 (0.75-1.33)∗	0.57 (0.36-0.85)∗∗††
<1.0	··	195 (42.3%)	105 (31.9%)	27 (47.4%)	63 (84.0%)
≥1.0	··	266 (57.7%)	224 (68.1%)	30 (52.6%)	12 (16.0%)
PLT, ×10^9^/L	125-350	218.5 (155.3-299.3)	226.5 (170.0-307.0)	229.0 (162.0-326.5)	164.0 (92.0-231.0)∗∗
NLR	NA	3.34 (2.06-6.41)	2.88 (1.85-4.42)	3.99 (2.43-6.29)∗∗	15.20 (8.08-28.57)∗∗††
PLR	NA	196.2 (142.3-295.5)	186.4 (141.1-267.1)	227.2 (142.9-314.4)	293.8 (155.2-430.0)∗∗
PT, s	9.2-15	11.80 (10.80-13.10)	11.50 (10.70-12.60)	11.70 (10.50-13.90)	14.43 (12.48-16.33)∗∗††
D-D, mg/L	0-0.55	0.95 (0.47-3.39)	0.72 (0.39-1.76)	1.70 (0.78-6.16)∗∗	3.17 (0.97-7.25)∗∗
Mean BG, mmol/L	3.9-6.1	5.92 (5.00-7.90)	5.70 (4.90-6.95)	6.63 (5.04-8.68)∗	8.10 (6.25-10.60)∗∗††
Normoglycemia	··	334/448 (74.6%)	262/317 (82.6%)	36/56 (64.3%)	36/75 (48.0%)
Hyperglycemia	··	114/448 (25.4%)	55/317 (17.4%)	20/56 (35.7%)	39/75 (52.0%)
HbA1c, %		6.00 (5.80-6.50)	6.00 (5.90-6.60)	5.90 (5.65-6.35)	5.60 (5.60-8.01)
Alb, g/L	40-55	33.70 (30.45-36.70)	34.30 (30.95-37.35)	33.20 (30.60-35.90)	30.70 (28.20-35.00)∗∗††
Cr, *μ*mol/L	57-97	67.00 (56.30-80.80)	65.90 (56.23-78.35)	65.20 (52.50-82.13)	74.20 (56.80-103.70)∗∗†
PCT, ng/mL	<0.05	0.10 (0.05-0.22)	0.06 (0.05-0.13)	0.06 (0.03-0.12)	0.24 (0.11-0.60)∗∗††
CRP, mg/L	0-4	18.30 (3.51-67.75)	13.69 (2.48-48.30)	14.81 (3.65-59.52)	93.40 (39.42-159.10)∗∗††
ESR, mm/h	0-20	40.0 (21.9-61.9)	40.0 (19.4-61.0)	31.0 (14.8-63.0)	44.0 (39.0-92.0)∗†

Note: Data represent the median (IQR), *n* (%) or *n*/*N* (%), where *N* is the total number of patients with available data. *P* values were determined using a *χ*^2^ test or the Mann–Whitney *U* test. NA, not applicable. COVID-19: coronavirus disease 2019; WBC: white blood cell count; NEU: neutrophil count; LYM: lymphocyte count; PLT: platelet count; NLR: neutrophil to lymphocyte ratio; PLR: platelet to lymphocyte ratio; PT: prothrombin time; D-D: D-dimer; BG: blood glucose; HbA1c: hemoglobin A1c; Alb: albumin; Cr: creatinine; PCT: procalcitonin; CRP: C-reactive protein; ESR: erythrocyte sedimentation rate. ∗*P* < 0.05; ∗∗*P* < 0.01, vs. the general group. †*P* < 0.05; ††*P* < 0.01, vs. the severe group.

**Table 2:** Clinical characteristics of COVID-19 patients grouped by comorbid diabetes status.

	Nondiabetic group (*n* = 366)			Diabetic group (*n* = 95)		
	Nondiabetics (*n* = 366)	Normoglycemia (*n* = 307)	Hyperglycemia (*n* = 46)	Diabetics (*n* = 95)	Normoglycemia (*n* = 27)	Hyperglycemia (*n* = 68)
Age, years	63.0 (53.0-69.0)	62.0 (52.0-69.0)	63.5 (55.0-70.0)	64.0 (54.0-70.0)	64.0 (61.0-72.0)	63.5 (55.0-70.0)
Male, %	50.5	50.8	52.1	56.8	44.4	61.8
BMI	23.40 (22.20-25.20)	23.58 (22.60-25.20)	23.49 (20.70-25.00)	23.23 (21.65-25.20)	22.68 (22.03-25.20)	24.80 (21.50-25.20)
Duration of hospitalization, days	12.00 (10.00-14.00)	12.00 (10.00-13.00)	13.50 (10.00-17.250)	16.00 (14.00-18.00)∗∗	14.00 (12.00-15.00)	16.00 (15.00-18.89)‡‡
Severity						
General	273 (74.6%)	244 (79.5%)	19 (41.3%)††	56 (58.9%)∗∗	18 (66.7%)	38 (55.9%)
Severe and critical	93 (25.4%)	63 (20.5%)	27 (58.7%)	39 (41.1%)	9 (33.3%)	30 (44.1%)
WBC, ×10^9^/L	5.58 (4.39-7.50)	5.50 (4.40-7.18)	6.80 (4.90-13.07)††	6.59 (4.80-9.00)∗∗	5.60 (4.61-7.81)	6.80 (5.00-10.43)‡
NEU, ×10^9^/L	3.85 (2.78-5.51)	3.71 (2.76-5.02)	5.49 (3.52-11.75)††	4.78 (3.21-6.95)∗∗	3.74 (3.10-5.57)	5.12 (3.34-8.07)‡‡
LYM, ×10^9^/L	1.13 (0.77-1.50)	1.15 (0.81-1.51)	0.82 (0.39-1.16)††	1.05 (0.67-1.57)	1.26 (0.69-1.84)	0.94 (0.65-1.32)‡
PLT, ×10^9^/L	220.0 (159.5-306.0)	223.0 (165.0-307.0)	191.0 (100.0-276.3)†	209.0 (148.0-280.0)	219.0 (139.0-256.0)	208.5 (148.3-289.0)
NLR	3.22 (2.00-5.90)	3.02 (1.95-4.93)	8.54 (3.56-18.67)††	4.39 (2.47-10.01)∗	2.79 (1.73-5.61)	5.04 (2.84-12.62)‡
PLR	199.1 (146.3-293.5)	196.2 (145.7-283.6)	231.1 (154.2-392.7)	189.9 (131.3-309.1)	142.7 (112.9-234.9)	198.4 (148.0-325.7)‡
Mean BG, mmol/L	5.54 (4.90-6.65)	5.35 (4.82-6.10)	9.09 (8.30-11.50)††	9.19 (7.69-12.30)∗∗	6.80 (6.30-7.24)	10.65 (8.73-13.81)‡‡
HbA1c, %	6.00 (5.70-6.40)	6.00 (5.70-6.40)	5.90 (5.60-6.32)	6.05 (5.80-8.35)	6.00 (5.80-6.30)	7.23 (5.75-9.14)
PCT, ng/mL	0.08 (0.05-0.21)	0.07 (0.05-0.18)	0.16 (0.07-0.38)††	0.13 (0.05-0.34)	0.06 (0.03-0.12)	0.18 (0.06-0.43)‡
CRP, mg/L	16.05 (3.17-64.00)	13.45 (2.80-51.98)	52.37 (18.06-135.96)††	32.20 (8.36-101.78)∗∗	27.33 (3.39-55.60)	46.94 (9.46-137.44)
ESR, mm/h	37.5 (19.0-61.5)	37.0 (18.4-60.9)	39.0 (25.0-108.8)	48.3 (38.0-64.1)∗	39.2 (21.0-60.0)	49.3 (39.0-75.3)

Note: Data represent the median (IQR) or *n* (%). *P* values were determined using a *χ*^2^ test or the Mann–Whitney *U* test. NA, not applicable. COVID-19: coronavirus disease 2019; BMI: body mass index; WBC: white blood cell count; NEU: neutrophil count; LYM: lymphocyte count; PLT: platelet count; NLR: neutrophil to lymphocyte ratio; PLR: platelet to lymphocyte ratio; BG: blood glucose; HbA1c: hemoglobin A1c; PCT: procalcitonin; CRP: C-reactive protein; ESR: erythrocyte sedimentation rate. ∗*P* < 0.05; ∗∗*P* < 0.01, vs. nondiabetic patients. †*P* < 0.05; ††*P* < 0.01, vs. the nondiabetic group, normoglycemia. ‡*P* < 0.05; ‡‡*P* < 0.01, vs. the diabetic group, normoglycemia.

## Data Availability

Data generated or analyzed during this study are included in this published article.

## Abbreviations

BG: Blood glucose
BMI: Body mass index
CI: Confidence interval
COVID-19: Coronavirus disease 2019
CRP: C-reactive protein
ESR: Erythrocyte sedimentation rate
FBG: Fasting blood glucose
HbA1c: Hemoglobin Ac1
IQR: Interquartile range
NLR: Neutrophil to lymphocyte ratio
OR: Odds ratio
PLR: Platelet to lymphocyte ratio
SARS-CoV-2: Severe acute respiratory syndrome coronavirus 2.
